# Editorial: Diversity, Equity, and Inclusion in Hernia Surgery

**DOI:** 10.3389/jaws.2024.14244

**Published:** 2025-02-07

**Authors:** Gabrielle H. van Ramshorst

**Affiliations:** ^1^ Department of Gastrointestinal Surgery, Ghent University Hospital, Ghent, Belgium; ^2^ Department of Human Structure and Repair, Ghent University, Ghent, Belgium

**Keywords:** diversity, equity, inclusion, inequity, gender-biased, gender gap, surgery, minorities

In this Special Issue of the Journal of Abdominal Wall Surgery, de Beaux shared his reflections on his inability to see issues about gender inequality. In his article, he shares reading tips regarding books which impacted on his views, sometimes bearing some uncomfortable truths. Diving deeper into Scottish history, the impressive stories of the Edinburgh Seven are described by Au and de Beaux.

The CanMeds roles state that as physicians, we need to demonstrate a commitment to patients by applying best practices [1]. Christoffersen and Henriksen found that more than half of the women with epigastric hernias in the Danish national database underwent suture-based repairs, even though mesh-based repairs reduce the rate of recurrence. Most groin hernias are found in men, therefore the article by Dahlstrand et al. on groin repairs in Swedish women adds to solving a knowledge gap. Only 19 out of 52 studies that included female patients showed separate results for women, highlighting an important focus for future study reporting. Following changes in guidelines, the proportion of endolaparoscopic surgery for groin hernia repairs (vs. open repair) has steadily risen over time in women, indicating growing adherence to guideline recommendations. Holland et al. explored racial and socioeconomic disparities in complex abdominal wall reconstruction referrals, as the equal access to minimally invasive surgery based on racial disparities has been a concern.

Some research questions will never be asked if female surgeons are not growing into principal investigators. And in order for them to climb the academic ladder, they need to be provided with opportunities for growth, mentoring and promoting from the early beginning of their careers. During training, female residents are perceived as needing more guidance and are offered less intraoperative autonomy [2–4]. Once in independent practice, women receive fewer referrals than men, especially from male colleagues [5]. This often results in less focused practices with fewer opportunities to build experience of performing complex procedures [6, 7].

Female surgeons least commonly performed the most lucrative surgical procedures [8]. Over a simulated 40 years career, female surgical specialists earn $2.5 million less than males after adjustment for factors such as hours worked, clinical revenue, type of practice and subspecialty, resulting in lower savings for retirement [9–11]. An American survey among over 25,000 academia, industry and government showed that all marginalised social groups earned less than white heterosexual males, with the latter granted more career opportunities, feeling more respected at work, experiencing less harassment and less likely to leave science [12].

A report from the Australian National Health Medical Research Council found that men were disproportionately awarded 23% more grants than women and received an additional $95 million in funding [13]. Women are less likely to be promoted even after adjusting for number of publications, amount of grant support, tenure vs. other career track, number of hours worked and specialty [14], and are less likely to become department chairs, as are specialists from non-white backgrounds [15]. In a randomised double blind study, applications with invented male names were rated as more competent and hirable by science faculty, given higher starter salaries and offered more mentoring whilst applications with invented female names were viewed as less competent [16].

Changing practice for the better requires a working culture that recognizes, supports and responds effectively to colleagues in need. Some barriers that women experience are invisible to others. In the operation room, if the surgeon’s gender differs from the primary gender composition of the rest of the surgical team, cooperation is higher, and conflict is lower [17]. Attending a (social event at a) conference can be a barrier to women and other minorities if they witnessed or experienced harassment inside and outside the hospital. Gender and racial based discrimination, verbal and physical abuse, and sexual harassment are reported at higher rates by women, with up to 65.1% of women reporting gender discrimination and 19.9% reporting sexual harassment [18]. A recently published systematic review by our research group describes the (additional) challenges that female surgeons face during pregnancy and early motherhood. [19] As members of the surgical community, we need to recognize and respond to unprofessional and unethical behaviours and some institutions have started to offer bystander training for developing this skill.

Creating an environment where females and underrepresented minorities are recognised as experts (not only as moderators) is an open opportunity for anyone who organises an educational event. If you are an invited speaker and the programme’s speakers are a poor representation of society: this is the time for you to speak up and promote others. The pharmaceutical and medical device industry is far behind, creating an industry payment gap -again in favour of male experts [20].

In 2023 I was awarded the American College of Surgeons Dr. Abdol and Mrs. Joan Islami International Guest Scholarship. One session at the annual meeting was dedicated to promoting women in leadership positions. And the following statement was shared: “A female leader must be competent, fearless and authentic.” I never heard a better description to fit Agneta Montgomery, a role model for so many female surgeons of my generation (Henriksen and Miserez). If you wish to be part of the solution: please find, mentor and promote more *Agnetas* to inspire the future generations of surgeons. Arrange a seat for them at the table where decisions are being made, as well as speaking time.

## References

[1] Frank JR Snell L Sherbino J, editors. CanMEDS 2015 Physician Competency Framework. Ottawa: Royal College of Physicians and Surgeons of Canada (2015).

[2] Hoops H Heston A Dewey E Spight D Brasel K Kiraly L. Resident Autonomy in the Operating Room: Does Gender Matter?. Am J Surg (2019) 217(2):301–5. 10.1016/j.amjsurg.2018.12.023 30580935

[3] Foley KE Izquierdo KM von Muchow MG Bastawrous AL Cleary RK Soliman MK. Colon and Rectal Surgery Robotic Training Programs: An Evaluation of Gender Disparities. Dis Colon Rectum (2020) 63(7):974–9. 10.1097/DCR.0000000000001625 32229780

[4] Joh DB van der Werf B Watson BJ French R Bann S Dennet E Assessment of Autonomy in Operative Procedures Among Female and Male New Zealand General Surgery Trainees. JAMA Surg (2020) 155(11):1019–26. 10.1001/jamasurg.2020.3021 32857160 PMC7450402

[5] Dossa F Zeltzer D Sutradhar R Simpson AN Baxter NN. Sex Differences in the Pattern of Patient Referrals to Male and Female Surgeons. JAMA Surg (2022) 157(2):95–103. 10.1001/jamasurg.2021.5784 34757424 PMC8581775

[6] Zhang B Westfal ML Griggs CL Hung YC Chang DC Kelleher CM. Practice Patterns and Work Environments That Influence Gender Inequality Among Academic Surgeons. Am J Surg (2020) 220(1):69–75. 10.1016/j.amjsurg.2019.10.029 31677781

[7] Chen YW Westfal ML Chang DC Kelleher CM. Contribution of Unequal New Patient Referrals to Female Surgeon Under-Employment. Am J Surg (2021) 222(4):746–750. 10.1016/j.amjsurg.2021.02.028 33685718

[8] Dossa F Simpson AN Sutradhar R Urbach DR Tomlinson G Detsky AS Sex-Based Disparities in the Hourly Earnings of Surgeons in the Fee-for-Service System in Ontario, Canada. JAMA Surg (2019) 154(12):1134–42. 10.1001/jamasurg.2019.3769 31577348 PMC6777399

[9] Jena AB Olenski AR Blumenthal DM. Sex Differences in Physician Salary in US Public Medical Schools. JAMA Intern Med (2016) 176(9):1294–304. 10.1001/jamainternmed.2016.3284 27400435 PMC5558151

[10] Whaley CM Koo T Arora VM Ganguli I Gross N Jena AB. Female Physicians Earn an Estimated $2 Million Less Than Male Physicians Over a Simulated 40-Year Career. Health Aff (Millwood) (2021) 40(12):1856–1864. 10.1377/hlthaff.2021.00461 34871074 PMC9910787

[11] Money Fit Women in Health Care. Fidelity Investments 2015 Report (2015). Available from: https://fidelity.com/bin-public/060_www_fidelity_com/documents/money-fit-women-in-health-care.pdf (Accessed December 21, 2024).

[12] Cech E. The Intersectional Privilege of White Able-Bodied Heterosexual Men in STEM. Sci Adv (2022) 8:1–14. 10.1126/sciadv.abo1558 PMC9200289 35704581

[13] Purton L Borger J. Is Australia’s Largest Medical Research Funding Body Doing Enough to Retain Women in STEMM? Available from: https://womensagenda.com.au/latest/is-australias-largest-medical-research-funding-body-doing-enough-to-retain-women-in-stemm/ (Accessed December 21, 2024).

[14] Jena AB Khullar D Ho O Olenski AR Blumenthal DM. Sex Differences in Academic Rank in US Medical Schools in 2014. JAMA (2015) 314(11):1149–58. 10.1001/jama.2015.10680 26372584 PMC4665995

[15] Kassam AF Taylor M Cortez AR Winer LK Quillin RC. Gender and Ethnic Diversity in Academic General Surgery Department Leadership. Am J Surg (2021) 221(2):363–368. 10.1016/j.amjsurg.2020.11.046 33261852

[16] Moss-Racusin CA Dovidio JF Brescoll VL Graham MJ Handelsman J. Science Faculty’s Subtle Gender Biases Favor Male Students. Proc Natl Acad Sci U S A (2012) 109(41):16474–9. 10.1073/pnas.1211286109 22988126 PMC3478626

[17] Jones LK Mowinski Jennings B Higgings MK De Waal FBM. Ethological Observations of Social Behavior in the Operating Room. Proc Natl Acad Sci U S A (2018) 115(29):7575–7580. 10.1073/pnas.1716883115 29967170 PMC6055187

[18] Hu YY Ellis RJ Hewitt DB Yang AD Ooi Cheung E Judith T Discrimination, Abuse, Harassment, and Burnout in Surgical Residency Training. N Engl J Med (2019) 381:1741–52. 10.1056/NEJMsa1903759 31657887 PMC6907686

[19] Noldus I Smisaert E Gijsels S Maeda Y van Ramshorst GH. Challenges in pregnancy and lactation among surgical residents and attendings: A systematic review. Surgery (April) 180, 109048. 10.1016/j.surg.2024.109048 39793416

[20] Storino A Vigna C Polanco-Santana JC Park E Crowell K Fabrizio A Disparities in Industry Funding Among Colorectal Surgeons: A Cross-Sectional Study. Surg Endosc (2022) 36:6592–6600. 10.1007/s00464-022-09062-8 35103858

